# Gene expression profiles of bladder cancers: evidence for a striking effect of *in vitro* cell models on gene patterns

**DOI:** 10.1038/sj.bjc.6600239

**Published:** 2002-04-22

**Authors:** V Dangles, V Lazar, P Validire, S Richon, M Wertheimer, V Laville, J-L Janneau, M Barrois, C Bovin, T Poynard, G Vallancien, D Bellet

**Affiliations:** Laboratoire d'Immunologie des Tumeurs, ESA 8067 CNRS, Faculté des Sciences Pharmaceutiques et Biologiques de Paris, Université Paris V – René Descartes, 4 avenue de l'Observatoire, 75006 Paris, France; Département de Biologie Clinique, Institut Gustave-Roussy, 39 rue Camille Desmoulins, 94805 Villejuif Cedex, France; Epigène, 63-65 boulevard Masséna, 75013 Paris, France; Service d'Anatomie-Pathologie, Institut Mutualiste Montsouris, 42 boulevard Jourdan, 75014 Paris, France; Pitié-Salpétrière, Assistance Publique-Hôpitaux de Paris, Service d'Hépatogastroentérologie, 47-83 boulevard de l'Hôpital, 75651 Paris Cedex, France; Service d'Urologie, Institut Mutualiste Montsouris, Paris, France

**Keywords:** bladder cancer, gene expression patterns, *in vitro* cell model

## Abstract

In order to assess the effect of *in vitro* models on the expression of key genes known to be implicated in the development or progression of cancer, we quantified by real-time quantitative PCR the expression of 28 key genes in three bladder cancer tissue specimens and in their derived cell lines, studied either as one-dimensional single cell suspensions, two-dimensional monolayers or three-dimensional spheroids. Global analysis of gene expression profiles showed that *in vitro* models had a dramatic impact upon gene expression. Remarkably, quantitative differences in gene expression of 2–63-fold were observed in 24 out of 28 genes among the cell models. In addition, we observed that the *in vitro* model which most closely mimicked *in vivo* mRNA phenotype varied with both the gene and the patient. These results provide evidence that mRNA expression databases based on cancer cell lines, which are studied to provide a rationale for selection of therapy on the basis of molecular characteristics of a patient's tumour, must be carefully interpreted.

*British Journal of Cancer* (2002) **86**, 1283–1289. DOI: 10.1038/sj/bjc/6600239 www.bjcancer.com

© 2002 Cancer Research UK

## 

Recently, with new genomic technologies, it has become possible to study the expression patterns of hundred of genes relevant to tumour development and progression. Gene expression profiles have often been assessed in human cancer cell lines in order to provide insights into biological events that take place during the malignant transformation of normal tissues (Kettunen *et al*, 2001) and to determine pharmacological implications of these genes in drug sensitivity or chemoresistance (Scherf *et al*, 2000; Wang *et al*, 2001). However, the billions of cells in the human body are more than a vast biochemical network of gene-encoded proteins. They are also physical entities with geometric dimensions and are therefore governed by the laws of macroscopic mechanics (Huang, 2000). Indeed, an individual normal cell, within the same biochemical milieu, will divide, differentiate or undergo apoptosis simply because of its external shape (Huang and Ingber, 1999).

Thus, the aim of this study was to investigate whether *in vitro* culture models displaying different cellular architectures influence gene expression profiles. A schematic view of our overall approach is shown in Figure 1

**Figure 1:**
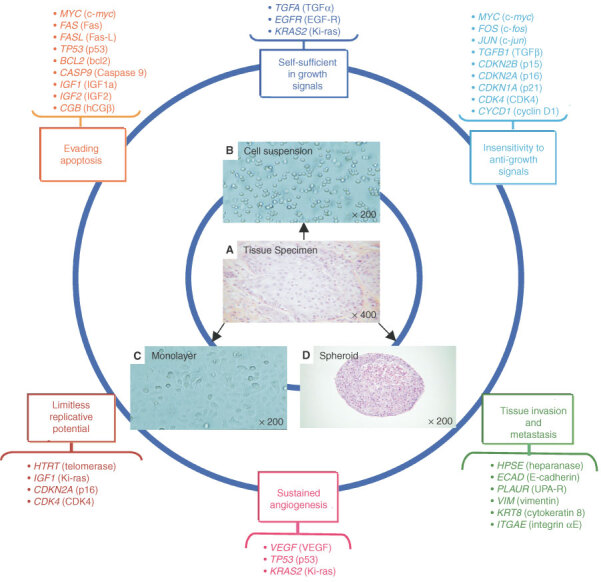
Design of the study. Expression profiles of genes involved in six essential alterations in cell physiology that collectively dictate malignant growth were studied in tissue specimens collected from three bladder tumours and *in vitro* cell culture models derived from these tumours and displaying three different architectural structures: one-dimensional (1-D) single cell suspensions, two-dimensional (2-D) monolayer cells or three-dimensional (3-D) multicellular spheroids. (**A**) Tumour specimen Tum 25, taken as representative. (**B**) Tum 25 cell suspension. (**C**) Tum 25 monolayer. (**D**) Tum 25 spheroid containing 10 000 tumour cells.

. Three bladder cancer cell lines established in our laboratory were used as an experimental model. These cell lines were studied as either one-dimensional (1-D) single-cell suspensions, two-dimensional (2-D) monolayers or three-dimensional (3-D) multicellular spheroids comprising 10 000 cancer cells. We focused on the expression of 28 genes well known to be involved in neoplastic transformation (Hanahan and Weinberg, 2000). These genes were quantified by real-time quantitative PCR both in the cell lines and in the parental bladder cancer tissue specimens collected from patients.

While pharmacological perspective currently tends to modify the target approach from a general to an individual treatment strategy, our data suggested that the *in vitro* model most precisely mimicking the *in vivo* situation must be determined for each patient and for each gene studied. Thus, in addition to expected individual patient variations, these data highlight the strong discordance between observations of gene expression in different *in vitro* models, providing the context in which current oncogenomic studies must be interpreted.

## MATERIALS AND METHODS

### Tissue specimens

Three tumour specimens, Tum 06, Tum 07 and Tum 25, were obtained from cystectomy of bladder carcinoma patients. After surgery, a tumour fragment was frozen in liquid nitrogen. Another fragment was collected in culture medium in order to establish tumour cell lines using previously reported protocols (Housseau *et al*, 1997). Cell lines were of tumour origin as assessed by phenotype analysis and karyology (Dr B Dutrillaux, Institut Curie, Paris, France). Moreover, the origin of tumour cell lines was certified by HLA typing (Dr G Semana, Etablissement Régional de Transfusion Sanguine de Bretagne-Est, Rennes, France). Histology and percentage of viable tumour cells in each specimen were determined by visual examination of adjacent sections stained with haematoxylin and eosin. Tum 06 was an adenocarcinoma and both Tum 07 and Tum 25 were transitional cell carcinomas. Tissue specimens from tumours Tum 07, Tum 25 and Tum 06 contained 80, 50 and 40% of tumour cells, respectively.

### *In vitro* cell culture

All types of cultures were cultured in the same medium consisting of RPMI 1640 with 4×10^−5^ M 2-β-mercaptoethanol, 2 mM L-glutamine, penicillin (100 U ml^−1^), streptomycin (100 μg ml^−1^), 1% sodium pyruvate, 0.1 mM non-essential amino acids, referred to as culture medium, supplemented with 10% heat-inactivated FCS and 10 μg ml^−1^ insulin (Sigma, Saint-Louis, MO, USA). Monolayers, suspensions and spheroids were obtained using the same thawed cell sample corresponding to the 26th, the 12th and the 11th passage of Tum 06, Tum 07 and Tum 25, respectively. Monolayer cultures were grown by seeding 0.5×10^6^ cells per 75 cm^2^ tissue culture flask. RNA extraction was performed on subconfluent cultures. Cell suspensions generated by trypsinization of tumour monolayers were resuspended in 10 ml of culture medium in a centrifuge tube for 1 h at 37°C in a humidified 5% CO_2_ incubator before RNA preparation. Multicellular spheroids were prepared in 96-well V-bottom culture dishes coated with poly-(2 hydroxyethyl metacrylate) (Sigma) as previously described (Dangles *et al*, 1997). After 5 days, spheroids contained an average number of 10 000 cells and were collected for RNA extraction. Trypan blue experiments performed on suspensions, monolayers or spheroids showed that cell culture conditions did not alter cell viability.

### Determination of mRNA levels using real-time PCR

Total RNA was isolated from either tissue samples or cultured cells by the acid-phenol guanidinium method, using RNAble (Bioprobe France). Total RNA concentration was determined at 260 nm and its quality was assessed by conventional gel electrophoresis. One μg of total RNA from each sample was reverse-transcribed and real-time quantitative PCR was conducted on an ABI prism SDS 7700 system (Perkin-Elmer Applied Biosystems Inc., Foster City, CA, USA), as previously described (Lazar *et al*, 1999). Briefly, oligonucleotide primers and TaqMan probes for analysed genes were designed for intron spanning using the computer program Primer Express (Perkin-Elmer Applied Biosystems Inc.) and sequences from the NCBI gene bank. Real-time quantitative PCR was achieved using a cDNA equivalent of 20 ng total RNA/50 μl per tube with the TaqMan® PCR Universal Master mix: 1× master mix (containing 5 mM MgCl_2_, 200 μM dA/C/G, 400 μM dU, 1.25 U AmpliTaq Gold® DNA polymerase, 2.5 U uracil N-glycosylase and glycerol), 100 mM TaqMan probe and 200 mM of each primer. To normalise for differences in the amount of total RNA added to the reaction, amplification of 18S ribosomal RNA was performed as an endogenous control. Primers and probes for 18S RNA were purchased from Perkin Elmer Applied Biosystems Inc.

To quantify the gene expression profile in each specimen we used the comparative threshold cycle (Ct) method according to the manufacturer's instructions. For each *in vitro* cellular model, a calibrator was constituted by the corresponding tumour specimen RNA. It was used as the 1×sample (or 100%) and all other levels (cultured cells) were expressed as an *n*-fold difference relative to this calibrator. The intra-assay coefficient of variations was less than 1%.

### Statistical analysis

Comparisons used general linear ANOVA models with the multiple comparison Bonferroni test. There were six measurements (six different total RNAs generated from six independent cultures) per gene (28 genes), per culture condition (three differing *in vitro* cell models) and per tumour tissue (three tumour specimens), leading to 1512 measurements. mRNA levels measured by real time quantitative PCR were transformed in logs to normalise distribution. To assess whether given gene expression for a given architecture and a given tumour was significantly different from *in vivo* measurement, the mean value of the six measurements was compared to one by the *t*-test, as all genes had been expressed as *n*-fold the calibrator. NCSS statistical software was used (Hintze JL. NCSS 97 User Guide. Number Cruncher Statistical Systems. Kaysville, UT, USA). To investigate which of the three *in vitro* cell models led to the gene profile closest to that observed *in vivo* for a specific gene and a specific tumour, the three values were compared by variance analysis and the multiple Bonferroni *t*-test if variance analysis was significant.

## RESULTS

### *In vitro* cell culture model and gene expression

Transitional cell bladder carcinoma fragments were obtained from patients after surgery (Figure 1A). We had previously established the two bladder carcinoma cell lines Tum 06 and Tum 07 from such tissue specimens (Housseau *et al*, 1997) and the additional autologous cell line Tum 25 was obtained with the same protocol. These three cell lines were studied *in vitro* as either (1-D) single cell suspensions (Figure 1B), (2-D) monolayer cells (Figure 1C), or (3-D) multicellular spheroids containing 10 000 cells (Figure 1D). *In vitro* cell architecture of spheroids was compared to that of tumour cells present *in vivo* within the tissue specimen. Histological features, including tight compaction, numerous mitoses and nucleolar atypia had led to the conclusion that the urothelial carcinoma cell microtumours obtained *in vitro* with spheroids were comparable to those observed upon cytological analysis of carcinoma abdominal spread.

**Figure 2:**
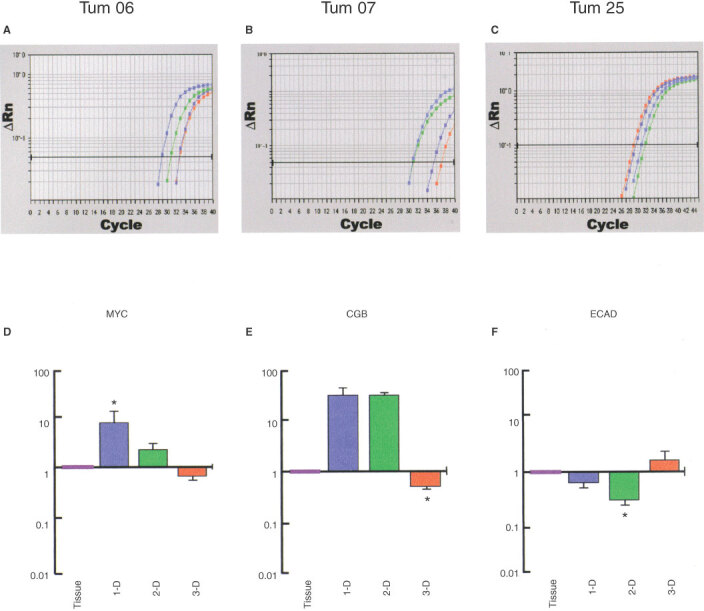
Impact of tumour architecture on gene expression. mRNA levels in tumour tissue specimens and in tumoral cell lines displaying 1-D, 2-D or 3-D structures were determined by real-time quantitative PCR. (**A**–**C**) Amplification plots; (**D**–**F**) expression levels in tissue (pink), in 1-D cell suspensions (blue), in 2-D monolayers (green), and in 3-D spheroids (orange), using each tumour specimen as the calibrator for the respective cell lines. Levels are expressed as an *n*-fold difference relative to the calibrator. All data are expressed as the mean±s.e.m. of at least two separate experiments carried out in triplicate. *, *P*<0.001, compared with other *in vitro* cell models (Bonferroni Multiple Comparison Test). (**A**, **D**) *MYC* gene encoding c-*myc* in tumour or cell line Tum 06. (**B**, **E**) *CGB* genes encoding hCGβ in tumour or cell line Tum 07. (**C**, **F**) *ECAD* gene encoding E-cadherin in tumour or cell line Tum 25, taken as representative.

The expression profiles of 28 genes known to be involved in malignant growth were studied in three bladder carcinoma fragments representative of the tumours in their *in* *vivo* architecture and in derived bladder cancer cells displaying different *in vitro* models. These genes were selected for their role in six essential alterations in cell physiology that collectively dictate malignant growth (Gilles *et al*, 1996; Bellet *et al*, 1997; Hendrix *et al*, 1997; Vlodavsky *et al*, 1999; Butler *et al*, 2000; Hanahan and Weinberg, 2000) (Figure 1). Real-time kinetic quantitative PCR was used to determine mRNA levels. To validate the real-time PCR method, standard curves for a given gene and for 18S ribosomal RNA were constructed from PCR products. 18S ribosomal RNA was used as standard, after comparison with *TBP* or *PPIA*, and was found to provide gene expression patterns similar to those provided with these transcripts as control genes (data not shown). Figure 2

shows the amplification plots (Figure 2A–C) and the expression levels (Figure 2D–F) of *MYC, CGB* and *ECAD* mRNAs, taken as representative, in Tum 06, Tum 07 and Tum 25 tissues and their associated cell lines.

**Figure 3:**
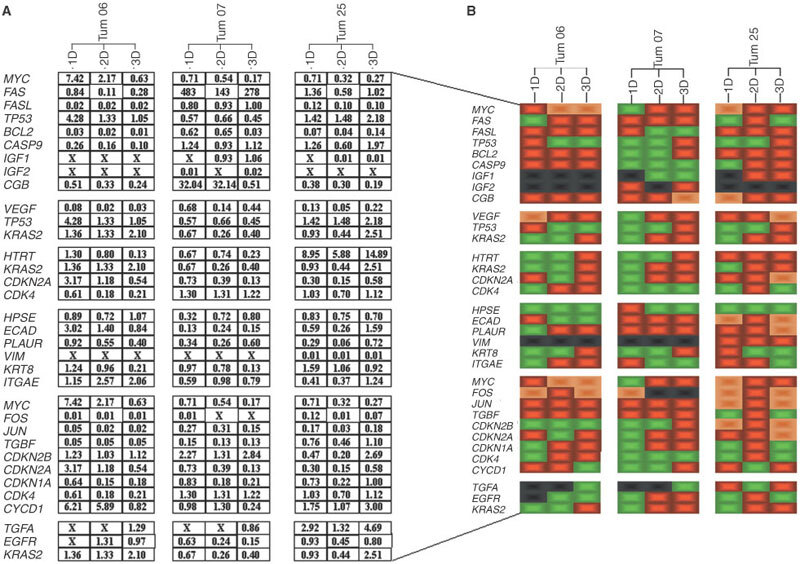
(**A**) Relative expression level of a given gene in a given tissue (mean value of six measurements) and expressed as *n*-fold the calibrator (tumour specimen RNA used as the 1×sample) : (x), no detectable transcripts. (**B**) Comparison of mRNA levels displayed by *in vitro* cell cultures with mRNA levels observed *in vivo*: black, no detectable transcripts; green, no statistical difference in mRNA levels between *in vitro* and *in vivo* cells; red, statistical difference in mRNA levels between *in vitro* and *in vivo* cells; orange, *in vitro* structure displaying mRNA levels which were closest to *in vivo* mRNA levels among the three significantly different models (*P*<0.03, Bonferroni Multiple Comparison Test).

Global mathematical analysis of the expression profile of the 28 key genes shows that the *in vitro* cell model has a striking effect upon gene expression: statistically significant differences between mRNA levels were consistently observed depending upon the 1-D, 2-D, or 3-D *in vitro* model of cell lines whatever the parental tumour. Indeed, there was a significant culture condition effect (*F*-Ratio=29; Degree Freedom=2; *P*<0.001). Interestingly, independent transcript analysis for each gene showed that this effect varied widely. Thus, a change in 1-D to 3-D cellular structure could, for example, lead to increased or reduced mRNA levels (Figure 2D–F). In addition to the influence of the *in vitro* model on gene expression, there was a significant tumour effect (*F*-Ratio=42; Degree Freedom=2; *P*<0.001) and a significant gene effect (*F*-ratio=171; Degree Freedom=27; *P*<0.001). For a given gene, variations in culture conditions could lead to either an increase or a decrease in mRNA levels, depending upon the tumour (Figure 3A

). Collectively, quantitative differences of between two-fold and 63-fold were observed for 24 out of 28 genes depending upon the tumour and the *in vitro* model: the 12 genes most up- or down-regulated by modified tumour culture are presented in Table 1

**Table 1:**
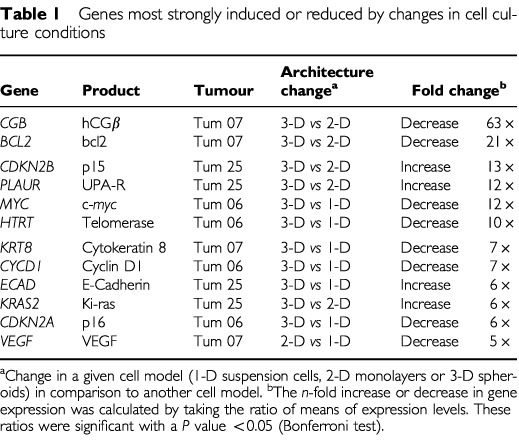
Genes most strongly induced or reduced by changes in cell culture conditions

.

### *In vitro* culture model and *in vivo* gene expression

In addition to this systematic study of the effects of *in vitro* culture conditions on gene expression, we were interested in determining whether a given *in vitro* tumour model closely mimics the *in vivo* gene expression profiles observed in cancer tissue. To conduct this investigation, we compared the expression profiles observed in the differing *in vitro* models with *in vivo* gene expression phenotypes. When all *in vitro* models displayed a significant difference in gene expression from the tumour specimen, we determined, when possible, the model which was closest to the *in vivo* situation. It is apparent that an *in vitro* cellular model displaying a gene expression profile similar to that observed *in vivo* varies with both the gene and the tumour (Figure 3B). As a representative example, we observed that a 3-D *in vitro* model leads to an *in vitro* gene profile similar to that displayed *in vivo* for *TP53* or *CDKN2B* only in Tum 06, for *FasL* only in Tum 07 or for *KRT8* only in Tum 25.

## DISCUSSION

Advances in molecular technology promise to yield quantitative measurements of thousands of mRNA levels at once, paving the way for such applications as molecular tumour profiling and precise tailoring of individual chemotherapy regimens. Most of these mRNA expression databases were obtained with cancer cell lines, particularly from the NCI-ACDS panel (Amundson *et al*, 2000; Scherf *et al*, 2000), to determine genes involved in drug sensitivity or cancer progression (Kettunen *et al*, 2001; Wang *et al*, 2001). Moreover, it is noteworthy that *in vitro* experiments traditionally use a 1-D or 2-D tumour cell model, whereas solid cancer cells display a 3-D geometry *in vivo*. Despite the fact that these basic parameters were not frequently taken into account, they have been demonstrated to have dramatic impact on biological responses of cells to differing stimuli (Mansbridge *et al*, 1994; Dangles *et al*, 1997; Huang and Ingber, 1999; Lang *et al*, 2001).

In this context, the present study was designed to determine the influence of *in vitro* cell models on gene expression profiles. With this aim, we took advantage of three bladder cancer cell lines established in our laboratory from parental tissue specimens and cultured in 1-D, 2-D or 3-D. We then studied genes implicated in six key alterations governing oncogenesis: self-sufficiency in growth signals, insensitivity to growth-inhibitory (antigrowth) signals, evasion of apoptosis, limitless replicative potential, sustained angiogenesis, and tissue invasion and metastasis.

In a first step, our findings demonstrated solid data of evidence for an impact of an *in vitro* model on gene expression. For 12 out of 28 studied genes, comparison of the transcript levels depending upon the *in vitro* culture condition showed rising ratio values from five to 63. It is noteworthy that 24 genes out of 28 presented variations superior to two, while, in a study based on conventional monolayers, only 28 of 1176 genes were altered >1.5-fold in five chemoresistant cell lines compared with the sensitive parental cell lines (Wang *et al*, 2001). Likewise, four out of 28 genes (14.2%) with an intensity ratio >3 were up- or down-regulated in the three bladder cancer cell lines in our study, whereas only 39 genes out of 588 (6.6%) were so modified in three malignant mesothelioma cell lines compared with a reference non tumoural cell line (Kettunen *et al*, 2001). Moreover, four genes had expression levels varying between 1.5- and two-fold but it is unclear whether these lower gene expression differences reflected true variations due to culture changes or gene-specific noises due to uncharacterized perturbations (Hughes *et al*, 2000). Interestingly, average values obtained for these ratios can be aligned with previous data obtained *in vivo* in normal and cancer cells of differing histological types by SAGE, array technologies or quantitative PCR (Zhang *et al*, 1997; Lal *et al*, 1999; Nacht *et al*, 1999; Sgroi *et al*, 1999). Collectively, it was shown that: (i) the vast majority of genes do not display differential expression; (ii) a limited subset of genes is differentially expressed at between 5- and 100-fold; and (iii) very few genes display gene expression differences higher than 100-fold between normal and cancer cells. With these data in mind, it is striking that changes in *in* *vitro* cellular models have a dramatic effect upon expression of key genes involved in malignant transformation, leading to differences attaining 63-fold. There are several possible explanations for the dramatic impact of *in vitro* cellular models. First, cell–cell interactions markedly differ between these three *in vitro* models, leading to changes in cellular geometry which could modify gene expression through mechanical forces or cell distortion (Huang and Ingber, 1999; Huang, 2000). Furthermore, variations in cell growth rates under the different culture conditions (data not shown) may participate in this phenomenon. Cell-to-cell contact might in any case either directly regulate gene expression, particularly the expression of genes involved in proliferation and apoptosis, or indirectly interact with gene expression through cell growth and viability functions.

We then studied whether an *in vitro* culture model, 1-D, 2-D or 3-D, might mimic the *in vivo* gene expression phenotypes. Our findings showed that *in vitro* culture conditions which display the gene profiles resembling those observed *in vivo* vary with both the gene and the patient. Indeed, 3-D was expected to be the most appropriate model for mimicking *in vivo* mRNA levels: paraffin sections of spheroids clearly showed that spheroid histoarchitecture was similar to *in vivo* tumour aggregates of disseminated carcinoma in ascites fluid, thereby suggesting the interest of spheroids as an *in vivo*-like model (Kunz-Schughart *et al*, 1998; Santini and Rainaldi, 1999). Interestingly, our results demonstrate that the 3-D spheroid is the most appropriate model for a limited subset of genes in a given tumour. However, it is highly likely that the heterogeneity of the tumour specimen has to be taken into account for a more thorough interpretation of these data. In this regard, it is noteworthy that the number of genes displaying an *in vitro* expression resembling that observed *in* *vivo* is higher for Tum 07 which contains the highest percentage of neoplastic cells (80%), in comparison to Tum 25 and Tum 06 (50 and 40%, respectively). As a representative example, no statistical difference in mRNA levels between *in vitro* cells in 1-D and *in vivo* cells was observed for 15 out 28 genes for Tum 07 in comparison to 11 and 10 out of 28 for Tum 06 and Tum 25, respectively. These results must be put in line with a recent analysis of mRNA profiles in prostate cancer which revealed a major division between cells grown *in vitro* and human tissue specimens, with highly divergent gene expression patterns (Welsh *et al*, 2001). The authors hypothesised that, because of the small number of genes with concordant expression in cell lines and malignant tissues, cell lines had lost many features that characterise prostate cancer *in vivo*. Nevertheless, the present data indicate that it is necessary to select, for a given gene and a given tumour, the *in vitro* model most closely mimicking *in vivo* expression of this gene. Moreover, in light of numerous reports underlining the disparity between mRNA transcript and protein levels (Simpson and Dorow, 2001), such mRNA expression studies should be supported by proteomic information to provide a complete picture of the *in vitro* model most closely mimicking the *in vivo* phenotype of the tumours.

Finally, these observations highlight complex regulation of the 30 000 human genes (Claverie, 2001) through the example of the dramatic influence of culture conditions on the expression of genes involved in malignant growth. Because we analysed a limited number of specimens, additional experiments using high throughput techniques are required to determine how commonly these genes are differentially expressed. Indeed, gene expression profiling using hundreds of genes might be useful not only for further characterising one of the three *in vitro* models of the tumour-derived cell lines, singling out their use as the most advantageous tumour model, but also for identifying genes associated with the phenotype of bladder cancers. However, our findings have important implications for the interpretation of data in the postgenome era, at the very time when gene expression is a prerequisite for designing and choosing an individual basis for specific therapies suited to individual patients.
